# Impact of medical TV shows on the surgical knowledge of non-healthcare students of Lahore, Pakistan

**DOI:** 10.1192/j.eurpsy.2023.2042

**Published:** 2023-07-19

**Authors:** S. Azeem, B. Mustafa, A. S. Ahmad, S. Rashid, M. Farooq

**Affiliations:** 1 King Edward Medical University; 2 Shalamar Medical and Dental College; 3 Fatima Memorial Hospital, Lahore, Pakistan

## Abstract

**Introduction:**

A popular genre of television shows is medical dramas. Although the primary objective of watching these shows is entertainment, acquiring medical knowledge is a passive by-product. Surgical procedures constitute a large part of the storyline of these shows. This could either serve as a source of medical knowledge or provide false information, the effect being especially important in individuals with no prior medical exposure.

**Objectives:**

This study aims to assess the impact medical TV shows can have on the surgical knowledge of non-healthcare students and the difference in knowledge between different demographic groups (among those with relatives in the medical community and those without).

**Methods:**

A cross-sectional study was conducted among the non-healthcare students of Lahore, Pakistan. A self-administered questionnaire was used containing socio-demographic factors (age, gender, educational discipline), history, and hours of medical TV shows watched. It also contained ten questions each with a score of 1 to assess surgical knowledge. Data was analyzed using SPSS v.26.

**Results:**

Among the 1097 respondents, 450 (41%) had a history of watching medical TV shows. The majority, 319 (29.1%), had seen these shows for < 24 hours. The mean score of all respondents was 5.79 out of a maximum score of 10. Respondents with a history of watching medical TV shows were more knowledgeable than those who did not (p < 0.001). Similarly, respondents with a history of watching more hours of medical TV shows were more knowledgeable than those who watched for a lesser number of hours (p < 0.001). Respondents with relatives in the healthcare profession were also more knowledgeable than those without (p = 0.049).

**Conclusions:**

If properly developed, while maintaining their primary entertainment value, medical TV shows can also be used as efficient learning tools. Quality controls must also be applied to minimize the risk of false information.

**Disclosure of Interest:**

None Declared

